# A moderated-mediation analysis of pathways in the association between Veterans’ health and their spouse’s relationship satisfaction: The importance of social support

**DOI:** 10.3389/fpsyg.2022.988814

**Published:** 2022-11-02

**Authors:** Christine Frank, Julie Coulthard, Jennifer E. C. Lee, Alla Skomorovsky

**Affiliations:** Director General Military Personnel Research and Analysis, Department of National Defence, Ottawa, ON, Canada

**Keywords:** relationship satisfaction, social support, caregiver burden, military, mental health, physical health, Veteran

## Abstract

**Introduction:**

Military personnel and Veterans are at increased risk of mental and physical health conditions, which can impact their families. Spouses often perform a vital role in caring for service members and Veterans facing illness or injury, which can lead to caregiver burden. In turn, this may contribute to relationship issues. Research suggests that ensuring that spouses are well supported can alleviate some of these negative effects. The current study examined whether social support received by spouses of newly released Veterans buffers the impact of Veterans’ health on caregiver burden, subsequently impacting spouses’ relationship satisfaction.

**Methods:**

Data were collected as part of the Canadian Armed Forces Transition and Well-being Survey. The sample included *N* = 595 spouses of Regular Force Veterans who released in 2016 with at least 2 years of service. We examined Veterans’ mental and physical health and spouses’ caregiver burden, social support, and relationship satisfaction. A moderated mediation model was tested using structural equation modeling.

**Results:**

There was a significant indirect association between Veterans’ health (both physical and mental) and spouses’ relationship satisfaction through caregiver burden. Furthermore, social support moderated the association, as evidenced by a weaker association between Veterans’ health and caregiver burden at low levels (−1SD) of social support compared to high levels (+1SD).

**Implications:**

Findings suggest additional efforts should be made to ensure sufficient support is provided to spouses, especially when they are caring for a service member or Veteran facing illness or injury, to strengthen their families’ well-being.

## Introduction

Evidence from past research has shown that deployment and exposure to combat heightens the risk of increased negative psychological sequela for service members and Veterans, such as posttraumatic stress disorder (PTSD), major depression, substance abuse, and functional impairments, along with an increased need for, and usage of, health care services (Hoge et al., 2006; Dekel and Monson, 2010; Boulos and Zamorski, 2013; Ebrahimzadeh et al., 2013). Notably, in one study, approximately one in six Regular Force Canadian Armed Forces (CAF) members reported symptoms of at least one of the following disorders: major depressive episode, panic disorder, PTSD, generalized anxiety disorder, and alcohol abuse or dependence. Respondents were also found to be nearly twice as likely to experience a mental health condition compared to the general Canadian population (Pearson et al., 2014). Being in the CAF also has a significant impact on a member’s physical health. The Health and Lifestyle Information Survey of Canadian Forces personnel reported that over a 12 month period, 32.3% of Regular Forces members reported sustaining a repetitive strain injury and 19.4% reported sustaining an acute injury that was severe enough to limit normal activities (Thériault et al., 2016). In addition, another study found that 50.6% of serving members and 67.1% of Veterans experienced chronic pain (i.e., pain that has lasted three to six months or longer; Perera et al., 2021). Each year, approximately 27% of CAF military releases are due to medical reasons (National Defence and Canadian Forces Ombudsman, 2016). In addition, a considerable number of Veterans who were not medically released may nevertheless live with health or other well-being problems (Thompson et al., 2016; Lee et al., 2020b).

### Provision of care and support among military/Veteran spouses

Spouses are considered to be central figures in establishing a secure base and maintaining family well-being throughout a service members’ military career (Green et al., 2013)—a role that may become especially important among military families facing additional challenges, such as illness, injury, or other disruptions. The presence of a significant mental or physical health condition impacts not only the service members or Veterans, but also their families (Link and Palinkas, 2013; Hachey, 2016). It can be particularly impactful for spouses, when they must step in as caregivers to provide care and support, often with direct effects to their own health and well-being (Ergh et al., 2003; Dekel et al., 2005; Christensen and Clinton, 2013; Ebrahimzadeh et al., 2013; Campbell et al., 2015). These family caregivers can take on extensive responsibilities covering a wide range of tasks, such as taking care of the finances, household maintenance, managing medical appointments and monitoring health status, transportation, performing or assisting with activities of daily living, and handling childcare (Hughes et al., 1999; Calhoun et al., 2002; Wakefield et al., 2012; Christensen and Clinton, 2013; Kratz et al., 2017). Spouses have also been shown to be an integral source of care support for Veterans as they transitioned out of the military, whether for medical reasons or not (Black and Papile, 2010; Skomorovsky et al., 2020; Lee et al., 2020b).

### Caregiving and relationship satisfaction

Caregivers may face various physical, emotional, cognitive, financial, and social challenges from caregiving (Baronet, 1999; Schulz and Martire, 2004; Skomorovsky et al., 2020) that can exceed the caregivers’ ability to manage the required demands. The necessity to take on additional responsibilities has been connected to reduced relationship satisfaction and increased marital distress (Dekel et al., 2005; Fredman et al., 2014). Spouses acting as a caregiver often have no choice but to make numerous adjustments (such as taking on additional responsibilities, previously shared between the spouses, accommodating behaviors, and role changes) leading to poor relationship adjustment (Dickson et al., 2010; Wakefield et al., 2012; Christensen and Clinton, 2013; Ebrahimzadeh et al., 2013). The shift in relationship dynamics as they assume the position of being a care provider rather than an intimate partner can lead to an increased risk of marital breakdown and decreased relationship satisfaction (Dekel et al., 2005; Renshaw et al., 2008; Karraker and Latham, 2015). Caregiver burden may be particularly high among those who are proving care for members with a diagnosed mental health disorder such as a traumatic brain injury (Brickell et al., 2019) or PTSD (Calhoun et al., 2002; Klarić et al., 2010).

Recent research suggests the perceived burden associated with caregiving is the mechanism by which members’ health impacts relationship satisfaction (Dekel et al., 2005; Skomorovsky et al., 2017). For example, among spouses of Veterans with PTSD, spousal distress was more closely associated with perceived caregiver burden than with the Veteran’s level of impairment (Dekel et al., 2005). Additionally, a study by Skomorovsky et al. (2017) found that caregiver burden mediated the link between the severity of members’ mental health condition (but not physical health condition) and divorce considerations in a sample of CAF members facing illness or injury and their spouses; however, the sample size was under 100, and the results should be replicated with a larger sample.

### Importance of social support

Researchers have emphasized the need to further examine the factors that moderate the links between health conditions, caregiver burden and family dysfunction (e.g., Link and Palinkas, 2013). One of the key buffers against the adverse effects of stress is believed to be social support (e.g., Uchino et al., 1996). According to the stress-buffering model, social support buffers the effect of stress on individuals’ well-being either by promoting alternative, more adaptive, coping strategies (Campbell-Sills et al., 2006; Henrich and Shahar, 2008) or by helping individuals redefine a situation as less threatening (Cohen and McKay, 1984)—reappraising the stressor in a more positive way (e.g., Bartone, 2006). Similarly, social support was found to play a critical role in the psychological well-being of military spouses: social support from family and partners were significant predictors of better psychological health and lower levels of depression among military spouses during their spouses’ deployment (Skomorovsky, 2014). Moreover, a number of studies have found a link between perceived social support and decreased caregiver burden (e.g., Thompson et al., 1993; Edwards and Scheetz, 2002; Rodakowski et al., 2012). In fact, a recent meta-analysis on the relationship between perceived social support and subjective burden among informal caregivers of adults or older adults found a moderate negative association between perceived support and subjective burden (Del-Pino-Casado et al., 2018).

While the literature has demonstrated that spousal caregivers may be at an increased risk for experiencing negative impacts, less research has been conducted to explore how social support may moderate the link between health, caregiver burden, and relationship satisfaction among the spouses of Canadian service members and Veterans. With a better understanding of the interrelationships between these factors, we could better inform efforts by policymakers and stakeholders in seeking to address the needs of military and Veteran spouses, especially when caring for Veterans facing illness or injury, to ensure appropriate supports are put in place to strengthen resilience among military families (Link and Palinkas, 2013).

### Current study

In the present study, we aimed to expand upon the findings of Skomorovsky et al. (2017) that perceptions of the severity of members’ mental health conditions were indirectly related to divorce considerations through caregiver burden. Specifically, we explored whether the extent of social support reported by spouses moderates the indirect association of their partner’s health with relationship satisfaction through caregiver burden. Further, given the prominent role played by spouses in the provision of care and support during the transition to civilian life (Black and Papile, 2010; Lee et al., 2020b; Skomorovsky et al., 2020), we examined these associations in a larger sample of spouses having recently undergone this transition.

### Hypotheses

1. There will be a significant indirect effect of member health on relationship satisfaction through perceived caregiver burden.
2. Social support will moderate the relationship between member health and perceived caregiver burden where the link between member health and perceived caregiver burden will be weaker when perceived social support is high and stronger when perceived social support is low.

## Materials and methods

### Survey sampling and data collection

Data were collected as part of the Canadian Armed Forces Transition and Well-being Survey (CAFTWS), which was conducted by Statistics Canada between April and June 2017 via computer-assisted interviews (Statistics Canada, 2018). The CAFTWS employed a stratified systematic random sampling approach, with the target sample frame drawn from the CAF human resources database. The first target population were all Regular Force Veterans who were released in 2016 with at least 2 years of service (730 or more days). CAF members who had been released for misconduct or unsatisfactory service were excluded from participation, owing to methodological challenges in reaching this subpopulation. Of primary interest in the present study, the second target population comprised the spouses or partners (hereafter referred to as “spouses” for the sake of simplicity) of the first target group, as identified through contact with the Veteran participants. Spouses participated in the CAFTWS by filling out a self-administered paper questionnaire, resulting in a sample of *N* = 595. Statistics Canada ensured that the project met ethical guidelines and required informed consent for participation. Additional information on the survey sampling and data collection can be retrieved elsewhere (Statistics Canada, 2018; Lee et al., 2020a).

### Measures

#### Veteran health

Veteran physical and mental health were each assessed using a single self-report item asking respondents “In general, would you say your [physical/mental] health is: poor, fair, good, very good, or excellent” (Ware et al., 1996). Higher scores indicate better perceived health. A meta-analytic review of the usage of the single item for mental health indicated the item correlated moderately with the Kessler Psychological Distress Scale (K10), the Patient Health Questionnaire, the mental health subscales of the Short-Form Health Status Survey, and increased health service utilization (Ahmad et al., 2014). Among respondents, 28.1% indicated their mental health was poor or fair, 23.7% indicated their mental health was good, 48.2% indicated their mental health was very good or excellent. Regarding physical health, 31.2% reported poor or fair physical health, 32.7% reported good physical health, 53.4% reported very good or excellent physical health. Self-rated physical and self-rated mental health were moderately correlated *r*(593) = 0.54, *p* < 0.001.

#### Spouse’s social support

Social support was assessed using the Social Provisions Scale (SPS) – short version (Cutrona and Russell, 1987). The scale includes 10 items that assess five types of social support (i.e., attachment, guidance, reliable alliance, social integration, and reassurance of worth). Participants were asked to rate the degree to which their social relationships provide each type of support on a 4-point Likert scale (from 1 “strongly agree” to 4 “strongly disagree”). Individual ratings were reverse-scored and summed for a total score with a possible range between 10 to 40, where higher scores represent higher perceived social support (*M* = 33.95, *SE* = 0.21). The SPS-short version has shown good reliability in the past (α = 0.84; Caron, 2013).

#### Spouse’s caregiver burden

In order to assess the degree of caregiver burden spouses felt as a result of the CAF Veteran’s physical or mental health, they were asked to complete the brief 12-item Zarit Burden Scale (Bédard et al., 2001). Participants were asked to indicate how often they experienced various feelings in relation to their spouse’s physical or mental health (e.g., do you feel you have lost control of your life since [care recipient’s illness]?) on a 5-point Likert scale (from 1 “never” to 5 “nearly always”).^1^ Individual ratings were summed for a total score with a possible range between 12 and 60 (*M* = 17.49, *SE* = 0.26), with higher scores indicating greater burden. The shortened scale has demonstrated excellent internal consistency in the past, with Cronbach’s alphas over .80 and .89 in the current study (Bédard et al., 2001; Martins Gratao et al., 2019).

#### Relationship satisfaction

Relationship Satisfaction was measured using three items from the Marriage Satisfaction Index (Norton, 1983). Specifically respondents were asked to rate their agreement on a 5-point Likert scale (from 1 “strongly agree” to 5 “strongly disagree”) with the following three statements “We have a good relationship;” “Our relationship is strong;” and “I am extremely happy with my relationship.” Individual ratings were given a mean score, for a total score with a possible range between one and five (*M* = 4.29, *SE* = 0.04), representing high degree of relationship satisfaction. The three items had good reliability (Cronbach’s Alpha = 0.93).

## Analysis

Sample weights generated by Statistics Canada were used to adjust for the complex sampling design and non-response. The spouse weights were applied to generate representative prevalence estimates for variables of interest as well as demographic and military characteristics. Proportions were generated to provide estimates for the prevalence of variables of interest. The 95% confidence intervals were generated for each of these estimates based on 1,000 bootstrap samples.

**Figure 1:**
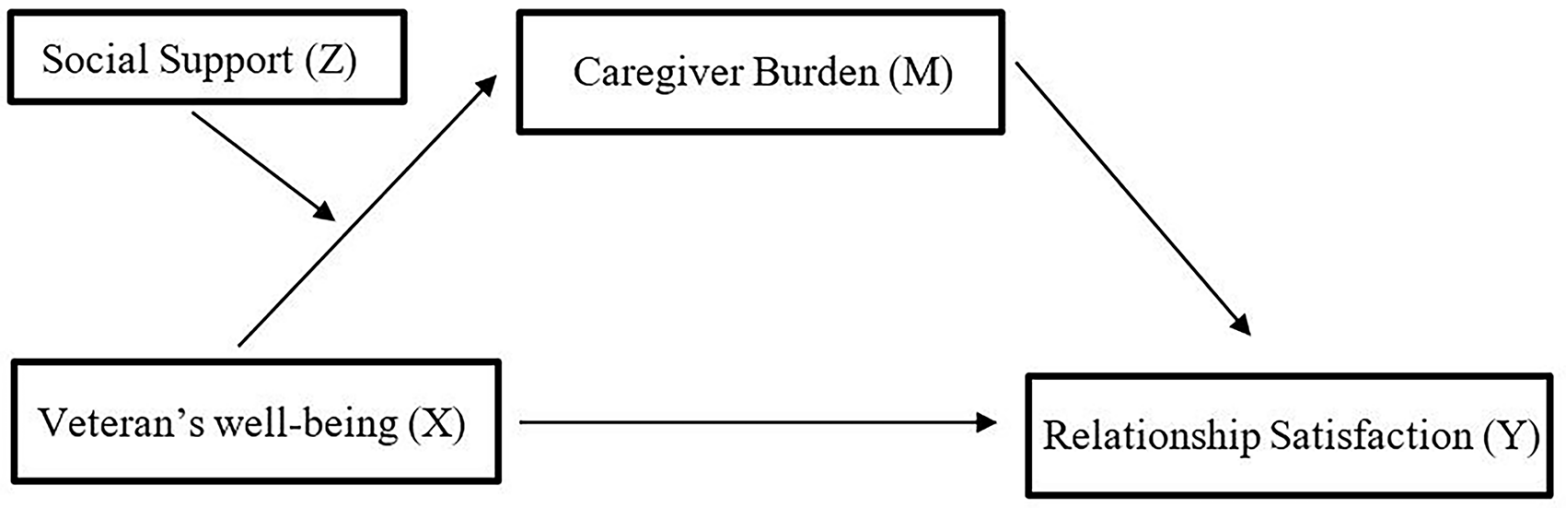
Model 7—moderated mediation model.

A moderated mediation analysis (see Figure 1) was conducted using Stata version 13.1. The first step was to test whether an indirect relationship existed between Veterans’ health and spouses’ relationship satisfaction through caregiver burden. The indirect effect was tested using the structural equation modeling (SEM) command with bootstrapping (1,000 replications). The nonlinear combination (nlcom) command bootstrapping (1,000 replications) was used to test whether the indirect association was significant. The nlcom command is used after SEM estimations to compute a point estimate, standard error, *p* value, and confidence interval for nonlinear combinations of the specified variables. The mediation models were tested independently for Veterans’ mental health and Veterans’ physical health.

Next, the moderated mediation was tested using the SEM command with bootstrapping (1,000 replications) where the interaction term between caregiver burden (M) and social support (Z) was added to the model. Next, the margins command was used to estimate the strength of association between Veteran’s well-being and spouse’s caregiver burden at low (-1SD), mean, and high (+1SD) levels of social support.

## Results

The current study aimed to assess whether spouses’ perceived caregiving burden mediated the association between the Veterans’ health and spouses’ relationship satisfaction, and whether this mediation was conditional on the amount of social support spouses received. The majority of participants were female (88.3%), over the age of 40 years old (70.8%) with approximately half (47.6%) reporting at least one dependent. Approximately two-thirds of respondents reported that their spouse (i.e., the Veteran) was their main source of support (i.e., companionship, assistance, or other support).^2^ More details on the socio-demographic and health information of participants are outlined in Table 1.

**Table 1:** Socio-demographic characteristics of Veterans and spouses (*N* = 595).

Demographic characteristics	Veterans	Spouses
	Proportion	Proportion
**Sex**		
Male	88.3%	11.7%
Female	11.7%	88.3%
**Age**		
20–29 years	6.8%	13.2%
30–39 years	22.5%	22.8%
40–49 years	30.6%	28.8%
50 years or more	40.2%	35. 3%
**Dependent(s) (<18 years) in household**		
Yes	45.2%	47.6%
No	54.8%	52.4%
**Education**		
Up to high school	41.7%	23.0%
Trade/college	27. 4%	41.1%
At least some university	30.9%	35.9%
**Source of support**		
Spouse		65.8%
Other		34.2%
**Military characteristics**		
**Years of service**		
2–5 years	3.8%	
6–9 years	9.4%	
10–19 years	22.2%	
20–34 years	46.1%	
35 years or more	18.6%	
**Rank**		
Junior NCM	31.9%	
Senior NCM	39.8%	
Junior officer	11.3%	
Senior officer	17.1%	
**Environment**		
Navy	19.7%	
Army	53.2%	
Air force	27.1%	
**Release type**		
Non-medical release	49.4%	
Medical release	50.6%	

### Indirect effects

First, the indirect associations between Veterans’ health (both mental and physical) and relationship satisfaction through caregiver burden was tested while controlling for spouses’ age, sex, main source of support, and whether they had dependents. As expected, the Veterans’ mental health significantly and negatively predicted caregiver burden (*B =* −6.66, *SE =* 1.55, *z =* −4.29, *p* < 0.001, 95% CI [−9.70; −3.62]); in turn, caregiver burden significantly and negatively predicted relationship satisfaction (*B =* −0.21, *SE = 0*.02, *z =* −12.00, *p* < 0.001, 95% CI [−0.24; −0.18]). The follow-up analysis supported a significant indirect association between Veterans’ mental health and relationship satisfaction through caregiver burden (*indirect effect* = 1.39, *SE* = 0.50, *z* = 2.78, *p* = 0.005, 95% CI [0.41; 2.38]).

In addition, Veterans’ physical health significantly and negatively predicted caregiver burden (*B =* −0.92, *SE =* 0.32, *z =* −2.88, *p* = 0.004, 95% CI [−1.55; −0.29]); in turn, caregiver burden also significantly and negatively predicted relationship satisfaction (*B =* −0.23, *SE =* 0.02, *z =* −14.32, *p* < 0.001, 95% CI [−0.26; −0.20]). The follow-up analysis supported a significant indirect association between Veterans’ physical health and relationship satisfaction through caregiver burden (*indirect effect* = 0.21, *SE* = 0.08, *z* = 2.72, *p* = 0.006, 95% CI [0.32; 0.87]).

The next step was to test whether social support interacted with Veterans’ health to predict caregiver burden. The interaction between Veterans’ mental health and social support predicting burden was significant (*B =* 0.08, *SE = 0*.04, *z =* 6.49, *p* < 0.001, 95% CI [0.01; 0.15]). The relationship between Veterans’ mental health and caregiver burden was stronger when social support was low (*B = −*3.07, *SE =* 0.26, *z = −*11.81, *p* < 0.001, 95% CI [−3.58; −2.56]) compared to the mean (*B = −*2.61, *SE =* 0.17, *z = −*15.77, *p* < 0.001, 95% CI [−2.93; −2.29]), with the association being weakest at high levels of social support (*B = −*2.15, *SE* = 0.26, *z = −*8.38, *p* < 0.001, 95% CI [−2.65; −1.65]).

Last, the interaction between Veterans’ physical health and social support predicting relationship satisfaction was marginally significant (*B =* 0.08, *SE =* 0.04, *z =* 1.95, *p* = 0.05, 95% CI [0.00; 0.17]). The relationship between caregiver burden and relationship satisfaction was stronger when social support was low (*B = −*2.60, *SE =* 0.3, *z = −*7.35, *p* < 0.001, 95% CI [−3.29; −1.90]), compared to the mean (*B = −*2.14, *SE =* 0.20, *z = −*10.56, *p* < 0.001, 95% CI [−2.54; −1.74]), with the association being weakest at high levels of social support (*B = −*1.68, *SE =* 0.26, *z = −*6.50, *p* < 0.001, 95% CI [−2.19; −1.17]).

## Discussion

The goal of the current study was to determine (1) whether there was a significant indirect effect of member health on relationship satisfaction through perceived caregiver burden; and (2) whether social support moderates the relation between member health and perceived caregiver burden where the link between member health and perceived caregiver burden will be weaker when perceived social support is high and stronger when perceived social support is low. Both Veterans’ mental and physical health were found to be indirectly associated with their spouses’ relationship satisfaction through caregiver burden. Additionally, perceived social support moderated this effect, as reflected by a weaker association between Veterans’ health and spouses’ caregiver burden at high levels of spouses’ social support and stronger association at low levels of spouses’ social support. The results of the study support and expand on the findings of Skomorovsky et al. (2017) that Veterans’ health indirectly influences relationship satisfaction through caregiver burden, by further providing evidence that social support acts as a buffer against the perceived burden of caring for a loved one, especially if they are facing illness or injury (Ong et al., 2018). However, in contrast to Skomorovsky et al.’s (2017) findings, an indirect effect was observed for both physical and mental health. This difference in findings may be due to Skomorovksy et al.’s use of spouses’ perceptions of members’ health rather than members’ perceptions of their own health, which they noted may be open to biases (e.g., the relationship between caregiver burden and spouses’ perceptions of members’ health may be bi-directional), or perhaps the sample size was too small to detect the indirect effects.

Our findings suggest that the presence of social support can mitigate the spill-over effects of service members’ or Veterans’ health problems onto spouses’ relationship satisfaction, thereby underscoring the importance of ensuring that spouses are well supported, especially when their partners are facing illness or injury. These findings have implications for institutions responsible for the development and delivery of support services for military and Veteran spouses (e.g., Military Family Resource Centers). Though there are currently programs and services available to families of military personnel and Veterans through CAF and Veterans Affairs Canada (VAC), there are also gaps. For example, for spouses of Veterans, VAC has established a caregiver zone, which includes a community of sharing online chat group, in addition to a caregiver coach support comprising of experts and health care professionals who can provide personalized guidance and advice. This resource may help spouses feel more socially integrated and supported through guidance, but services or programs providing other forms of social support (e.g., tangible support) may be lacking. Indeed, there have been increasing calls to expand support services for spouses who are having to provide additional care or support to their partner either due to illness or injury or during the transition from military to civilian life in recent years (Cramm et al., 2020; Keeling et al., 2020; Corry et al., 2022). Exploring which services and programs are available, or can be adapted, to best serve the spouses of service members and Veterans may prove to be a worthwhile direction in future research.

### Strengths and limitations

Some limitations of the study should be noted. First, the study only included a subjective measure of caregiver burden. However, the literature emphasizes the importance of the subjective burden, highlighting the self-perceived impact and negative feelings associated in caregivers as they fulfill these objective caregiving functions (Calhoun et al., 2002; Christensen and Clinton, 2013; Bayen et al., 2014; Cao and Yang, 2020).

Another limitation is that the study only includes CAF Veterans who had recently transitioned out of the CAF (for a variety of reasons), and not active service members. As such, the generalizability of the study may be limited to recently released personnel, and may not necessarily extend to active military personnel and their spouses.

Another important limitation is the cross-sectional design of this study, which does not make it possible to examine a causal relationship between variables, such as between perceptions of social support and psychological well-being. For example, it is possible that individuals who have poorer psychological health are subsequently less likely to engage in social networking and not seek social support or perceive the social support as unavailable even when there is an availability of social support (e.g., Sharkansky et al., 2000). Future research may consider using a longitudinal approach to help determine the directionality (or bi-directionality) of the relationships identified in the current study.

A major strength of the study is that we were able to include Veterans’ own perceptions of their mental and physical health rather than their spouse’s perceptions of their health, which may be more biased, in our statistical models. This allowed us to rule out the possibility that our assessment of Veterans’ health status was influenced by their spouses’ perceptions of caregiver burden. Continued work in the area should consider utilizing this mixed method approach.

## Conclusion

This study addressed a gap in the current literature by examining the buffering effect of social support on the pathway between Veterans’ health and spouses’ relationship satisfaction through caregiver burden. Results were consistent with the hypotheses and previous research findings, demonstrating that social support can buffer the negative effects of illness or injury and caregiver burden on relationship satisfaction among spouses of Veterans. Military and Veteran organizations must continue to expand on, and provide, programs and services to address the unique needs of military and Veteran spouses and facilitate their access different components of social support.

## Data availability statement

The data analyzed in this study is subject to the following licenses/restrictions: The dataset is the property of, and retained by, Statistics Canada. Data can be accessible at Research Data Centers in various locations across Canada by researchers from Canadian institutions and Federal departments through a formal application process. Requests to access these datasets should be directed to statcan.dad-apu-dad-uta.statcan@statcan.gc.ca.

## Ethics statement

The studies involving human participants were reviewed and approved by All project proposals within Statistics Canada are reviewed and approved by their internal data ethics secretariat. The patients/participants provided their written informed consent to participate in this study.

## Author contributions

CF conducted the analysis and wrote the methods and results sections as well as general editing. JC wrote the introduction. JL wrote the discussion. AS contributed to the introduction and discussion content. All authors contributed to the article and approved the submitted version.

## Conflict of interest

The authors declare that the research was conducted in the absence of any commercial or financial relationships that could be construed as a potential conflict of interest.

## Publisher’s note

All claims expressed in this article are solely those of the authors and do not necessarily represent those of their affiliated organizations, or those of the publisher, the editors and the reviewers. Any product that may be evaluated in this article, or claim that may be made by its manufacturer, is not guaranteed or endorsed by the publisher.

## Author disclaimer

This manuscript presents the opinions of the authors and does not necessarily reflect the official position of the Department of National Defence or Canadian Armed Forces.

## Footnotes

^1^All spouses were invited to complete this scale, regardless of the Veteran’s health status or reason for being released from the CAF. In the survey questionnaire, it was noted that the questions might not apply to all participants, but it was necessary to ask the same questions of everyone.

^2^Other sources of support include: parent, sibling, son/daughter, extended family member, friend, supervisor, co-worker, or health professional.
